# Drosophila *Clueless* Is Highly Expressed in Larval Neuroblasts, Affects Mitochondrial Localization and Suppresses Mitochondrial Oxidative Damage

**DOI:** 10.1371/journal.pone.0054283

**Published:** 2013-01-16

**Authors:** Aditya Sen, Vanessa T. Damm, Rachel T. Cox

**Affiliations:** 1 Department of Biochemistry and Molecular Biology, Uniformed Services University, Bethesda, Maryland, United States of America; 2 Center for Neuroscience and Regenerative Medicine, Uniformed Services University, Bethesda, Maryland, United States of America; Cardiff University, United Kingdom

## Abstract

Mitochondria are critical for neuronal function due to the high demand of ATP in these cell types. During Drosophila development, neuroblasts in the larval brain divide asymmetrically to populate the adult central nervous system. While many of the proteins responsible for maintaining neuroblast cell fate and asymmetric cell divisions are known, little is know about the role of metabolism and mitochondria in neuroblast division and maintenance. The gene *clueless* (*clu*) has been previously shown to be important for mitochondrial function. *clu* mutant adults have severely shortened lifespans and are highly uncoordinated. Part of their lack of coordination is due to defects in muscle, however, in this study we have identified high levels of Clu expression in larval neuroblasts and other regions of the dividing larval brain. We show while mitochondria in *clu* mutant neuroblasts are mislocalized during the cell cycle, surprisingly, overall brain morphology appears to be normal. This is explained by our observation that *clu* mutant larvae have normal levels of ATP and do not suffer oxidative damage, in sharp contrast to *clu* mutant adults. Mutations in two other genes encoding mitochondrial proteins, *technical knockout* and *stress sensitive B*, do not cause neuroblast mitochondrial mislocalization, even though *technical knockout* mutant larvae suffer oxidative damage. These results suggest Clu functions upstream of electron transport and oxidative phosphorylation, has a role in suppressing oxidative damage in the cell, and that lack of Clu’s specific function causes mitochondria to mislocalize. These results also support the previous observation that larval development relies on aerobic glycolysis, rather than oxidative phosphorylation. Thus Clu’s role in mitochondrial function is not critical during larval development, but is important for pupae and adults.

## Introduction

Mitochondria are the main ATP supplier in most cell types. Mitochondrial numbers are maintained through mitochondrial fission and replication, since cells cannot make these organelles de novo [1], [2]. Thus during mitosis, mitochondria must localize and segregate normally from mother to daughter cell. In addition, disruptions to mitochondrial function are involved in a myriad of diseases that affect the nervous system because synapse function relies on large numbers of mitochondria and a steady supply of ATP [3].

**Figure 1:**
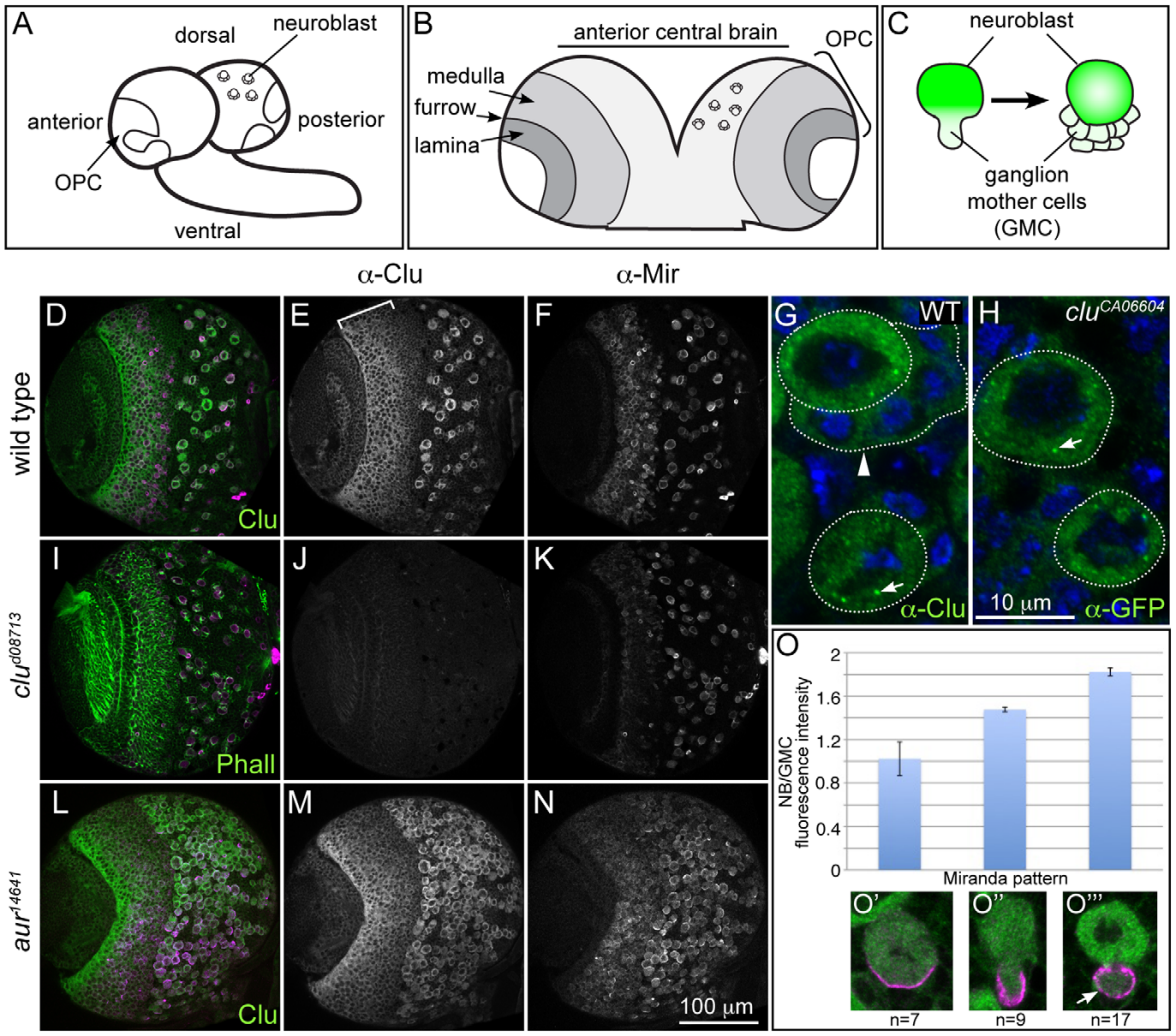
Clu protein is highly expressed in NBs. (A–C) Schematics of the larval brain and NB division. A) The larval brain, showing the outerproliferative center (OPC) and central brain neuroblasts. B) The OPC consists of the medulla and the lamina separated by the laminar furrow. C) Neuroblasts (NB) repeatedly divide asymmetrically to produce daughter ganglion mother cells (GMCs) that go on to differentiate and populate the adult central nervous system. (D–F) Wild type Clu protein localization in the third instar brain. D) Clu and Mir both label the same cells. E) Clu is highly expressed in the cytoplasm of large cells in the central brain, as well as medullar neuroblasts (bracket). F) Mir antibody labeling central brain NBs and medullar neuroblasts. G) WT NBs labeled with Clu antibody. NBs (round dotted outline) contain cytoplasmic Clu, as well as distinct Clu particles (arrow). Daughter GMCs (arrowhead, outline) have markedly lower amounts of Clu. H) *clu^CA06604^* Clu GFP-trap NBs labeled with anti-GFP antibody also show cytoplasmic Clu and Clu particles (arrow). (I–K) *clu^d08713^* mutant brains lack any detectable Clu expression. I) *clu^d08713^* mutant brain labeled with anti-Clu antibody, Mir and phalloidin. J) *clu^d08713^* lacks Clu expression but has normal Mir expression (K). (L–N) Clu is expressed in ectopic NBs formed in *aur* mutant larval brains. *aur^14641^* mutant brains have a greatly increased number of Mir positive NBs (N). These NBs also label with Clu antibody (L and M). (O) Clu protein levels are decreased in GMCs compared to NBs, even when the GMC is still connected to the NB. (O’–O’’’) Clu and Mir antibody labeling denotes the NB cell cycle stage examined for Clu expression. anti-Clu - green in D, G, H, L, O’–O’”, white in E, J, M. anti-Mir- magenta in D, I, L, O’–O’”, white in F, K, N. Phalloidin (green), I. Error bars: N for D–F, I–K, L–N. H for G and H.

In the Drosophila larval brain, neuroblasts (NBs) are the stem cell-like cells that undergo asymmetric divisions to give rise to daughter cells that will populate the adult CNS (Fig. 1A). Genes that are specifically expressed in neuroblasts generally fall into two categories: those that establish the asymmetric cell division (Fig. 1C), and those involving developmentally timed changes in transcription factors, known as temporal factor switching (reviewed in [4], [5], [6], [7]). Proteins that set up the asymmetric NB cell division along the apicobasal axis involve membrane bound proteins localized in discreet domains that orient the mitotic spindle. In contrast, temporal factor switching is due to intrinsic regulation of NB divisions by transcription factor cascades. While much is known about these two mechanisms controlling NB fate and division, little is known about the expression levels of genes underlying metabolism in NBs, and what role they may play during larval CNS development. In addition, how mitochondria contribute to larval NB maintenance and division has not been characterized during the NB cell cycle.

*clueless* is a highly conserved gene important for maintaining mitochondrial function and localization. *clu* mutant flies are male and female sterile and mitochondria in female germ cells are severely mislocalized [8]. This mitochondrial mislocalization is conserved as mutations in the *clu* homologs in yeast, Dictyostelium and Arabidopsis also mislocalize mitochondria [9], [10], [11], [12]. Few *clu* mutant adults eclose, and those that do are highly uncoordinated and die quickly [8]. Because *clu* adults are so uncoordinated, and mitochondria are critical for neural function, we examined if lack of *clu* caused abnormalities during brain development. We find mitochondria in wild type larval NBs undergo stereotypical location changes during the cell cycle. For most of the cell cycle, they remain small and dispersed, likely because the NB cell cycle is so rapid. In *clu* mutant NBs, mitochondria are severely mislocalized, in contrast to mutations in two other mitochondrial proteins, *technical knockout* (*tko*) and *stress sensitive B* (*sesB*). Furthermore, Clu is highly expressed in dividing cells in the larval brains, including larval NBs and medullar neuroblasts. Surprisingly, *clu* mutant larvae develop normally, and there is not a large effect on larval brain development. This is likely because in *clu* mutant larvae, ATP levels are at wild type levels and the larvae do not experience oxidative stress. In contrast, *clu* mutant adults have greatly diminished amounts of ATP and experience increased amounts of oxidative stress. These results show that Clu function is required in adults in order to generate normal amounts of ATP and suppress oxidative damage, but does not appear to be critical for larval development. Because Clu’s mitochondrial function is not necessary in larvae, our studies support the observation that larval metabolism involves aerobic glycolysis, instead of relying on oxidative phosphorylation [13].

## Materials and Methods

### Fly Stocks

The following stocks were used for experiments: *clueless^d08713^*/CyO Act GFP [8], *aurora^14641^*/TM6b [14], *tko^25t^* [15], and *mtATP6^1^*; *sesB^1^* [16], [17]. *SOD2* ^Δ*2*^/CyO Act GFP and Df(2)Jp4/CyO were obtained from the Bloomington Drosophila Stock Center. For wild type, *y^1^ w^67g23^* was used. Flies were reared on standard cornmeal fly media at 22° or 25°C.

### Hatching, Pupation, Eclosion and Lifespan Measurements

For hatching experiments, 100 embryos of each genotype were placed on a molasses agar plate. The number of larvae were counted after 24 hours and were scored for GFP. Each genotype was performed in triplicate. For pupation and eclosion, twenty *clu^d08713^* (GFP−) and twenty *clu^d08713^*/CyO Act GFP (GFP+) first instar larvae were collected and placed in vials. At the onset of pupation, we recorded the number of pupae each day. Once they began eclosing, we recorded the number of adults each day. Each genotype was performed in triplicate. For lifespan measurements, twenty 0–4 hr flies (ten male and ten female) of the appropriate genotype were placed in unyeasted vials. The number of dead flies was counted each day, and the flies were transferred every two days. Each genotype was performed in triplicate.

### Immunofluorescence and Western Blotting

Third instar larval brains were dissected in room temperature (RT) in Grace’s Insect Medium (modified) (BioWhittaker, Lonza, Cologne, Germany). Brains were fixed for 25 minutes in 4% paraformaldehyde and 20 mM formic acid solution (Sigma) made in Grace’s. For tubulin staining, 1 mM EGTA was included. Tissues were washed 3 times, 10 minutes each with antibody wash buffer (1× PBS:0.1% Triton X-100∶1% BSA) and were incubated in primary antibody over night at 4°C. They were then washed 3×10 minutes and incubated overnight at 4°C in secondary antibody. For actin labeling, rhodamine phalloidin (1∶200, Molecular Probes, Invitrogen) was added with the primary antibody. After washing 3×10 minutes, DAPI was added for five minutes then removed, then Vectashield (Vector Laboratories, Inc.) was added. The following primary antibodies were used: guinea pig anti-Clu N-terminus [8], mouse anti-Complex V alpha subunit (1∶1000, Mitosciences, Inc), rhodamine phalloidin (1∶200, Molecular Probes, Invitrogen), rat anti-Miranda (1∶100, gift of Dr. Chris Doe), Tubulin cocktail: 1∶1:1, AA4.3:AA12.1:E7 (1∶50, Developmental Studies Hybridoma Bank, University of Iowa), rabbit anti-phosphohistone H3 (1∶1000, Abcam, Inc.). The following secondary antibodies were used: anti-mouse IgG_2b_ Alexa 488, anti mouse IgG_1_ Alexa 568, anti-guinea pig Alexa 488 (Molecular Probes, Invitrogen). Brains were imaged using a Zeiss 710 confocal microscope and 63× Plan Apo NA 1.4 lens. Western blots were performed as described in [8].

### Clu Protein Quantification

To determine the amount of decrease in Clu protein in GMCs compared to NBs, third instar brains labeled with anti-Clu, anti-Mir antibodies and DAPI were imaged using the confocal. We outlined a region of interest (ROI) in the apical NB cytoplasm. Using ImageJ, we counted the total fluorescent intensity. Using the same ROI, we counted the fluorescent intensity in the emerging or separated GMC and divided the fluorescent intensity in the NB by the intensity in the GMC. For the control, we counted the intensity in an apical region, and a basal region in NBs expressing a strong Mir crescent.

### Aconitase Activity Assay

Approximately 20 flies or 35 larvae were homogenized in 90 µl extraction buffer (50 mM Tris–HCl, pH 8.0, 0.6 mM MnCl_2_, 2 mM citric acid, 1% NP-40, 1× protease inhibitor cocktail (Roche)) and incubated on ice for 30 min. The homogenate was spun at 13,000 g for 5 minutes at 4°C. The supernatant was subsequently transferred and total protein concentrations determined by Bradford assay. Normalized samples were blotted using a pipet onto a cellulose acetate membrane (Cellogel, Accurate Chemicals, NJ) and subjected to electrophoresis at 150 V for 40 minutes at 4°C in running buffer (20 mM potassium phosphate (pH 7.8), 3.6 mM citrate) to separate mitochondrial and cytoplasmic aconitase pools. After electrophoresis, the membrane was dipped in staining solution (100 mM potassium phosphate, (pH 6.5), 1 mM NADP^+^, 25 mM MgCl_2_, 2 mM cis-aconitic acid, 0.5 mg/ml 2,3-bis-(2-methoxy-4-nitro-5-sulfenyl)-2H-tetrazolium-5 carboxanilide disodium salt (XTT) or MTT, 0.3 mM phenazine methosulfate, 5 units/ml isocitrate dehydrogenase) for 10 minutes. After two quick washes in running buffer, the membrane was scanned for imaging and quantification, for which three blots were used. A representative blot is shown in Figure S4. For reactivating the mitochondrial aconitase, the sample was exposed to 50 mM DTT and 1/20 volume ferrous ammonium sulfate (Biovision, Inc.) before being run on the membrane.

### ATP Assay

30 larvae or 22 adults were homogenized in 300 µl or 200 µl, respectively, extraction buffer (100 mM Tris-Cl, pH 8.0, 4 mM EDTA, pH 8.0; 6 M guanidine hydrochloride), boiled for 4 minutes, then centrifuged at 8000 g for 5 minutes at 4°C. The protein concentration of the samples was determined using a Bradford assay. The ATP concentration was determined using an ATP Determination Kit (Molecular Probes, Invitrogen) according to the manufacturers directions. 100 µl assays were performed in a 96 well plate and the luminescence was measured using a Biotek Synergy H1 luminometer. Each sample was processed in duplicate and read in duplicate. The amount of ATP was normalized against protein concentration.

## Results

### Clu Protein is Highly Expressed in Larval Neuroblasts

Adults lacking Clu protein are highly uncoordinated, and within a day or two after eclosion are unable to climb or fly [8]. While this is partly due to defects in their muscle [8], we wanted to investigate the role of Clu on brain development. In order to do this, we analyzed Clu’s localization pattern in the third instar larval brain (Fig. 1A). Clu protein is highly and specifically expressed in the cytoplasm of large cells located in the central brain (Fig. 1D, E, G, H). To confirm these large cells are neuroblasts (NB), we co-labeled with the NB specific marker Miranda and find they label the same cells (Fig. 1D, F, O’,). To determine if Clu levels are also high in ectopically created NBs, we examined *aurora* (*aur*) mutant larval brains. *aur^14641^* mutant larval brains contain excessive numbers of NBs because they incorrectly divide symmetrically [14], [18]. In *aur^14641^* mutant larval brains, Clu still labels all the Mir positive cells in the central brain indicating Clu expression is specific to NB cell fate (Fig. 1 L–N). Clu protein in female germ cells is highly expressed in the cytoplasm, as well as in large, discrete particles [8]. Upon closer inspection and after decreasing the brightness while imaging, this is also the case in NBs. There are high levels of Clu found uniformly in the cytoplasm, however, there are particles as well (Fig. 1G, H, arrows). Clu expression is not restricted to central brain NBs, but is also high in other proliferative zones of the larval brain (Fig. 1B). These include the medullar neuroblasts of the outer proliferative center, the inner proliferative center, and the NBs in the ventral nerve chord (Fig. 1A, B, D, E, Fig. S1). Subcellular Clu particles are also present in these cells types (Fig. S1D).

Clu remains high in the cytoplasm of the NB, but is at much lower levels in the daughter ganglion mother cells (GMCs) (Fig. 1G, arrowhead, 1O’”, arrow). In order to investigate the timing of Clu decrease, we measured Clu fluorescent intensity during the NB cell cycle. As the GMC is forming during anaphase, Clu protein is already decreasing (Fig. 1O, green). Immediately after the GMC has fully separated from the NB, its Clu expression decreases by nearly half (Fig. 1O’”). This suggests that either less Clu is segregated to the GMC, or there is a mechanism in place to initiate protein degradation even when the NB and GMC cytoplasms are connected during anaphase.

Although Clu is highly expressed in neuroblasts, *clu* mutants do not have any gross morphological defects in brain development (Fig. S2A, B). Mir labeling is normal in *clu^d08713^* mutants (Fig. 1K), and there are the same number of NBs in *clu^d08713^* mutant brains compared to wild type (Fig. S2E, Supplementary Materials and Methods), indicating Clu protein is not necessary for stem cell maintenance. In addition, actin-rich axonal projections from the GMC progeny are present (data not shown), and anti-Prospero antibody appears to label differentiating neurons in a normal pattern (Fig. S2C, D). The normal NB number in *clu* mutants could be attributed to the large wild type maternal contribution, however larvae maternally and zygotically mutant for *clu* can still eclose and do not exhibit a worse NB phenotype (see below, Fig. S3). Taken together, these data indicate that Clu function is not important for stem cell maintenance and differentiation during larval brain development.

### Neuroblast Mitochondria are Distinct, Small Spheres

**Figure 2:**
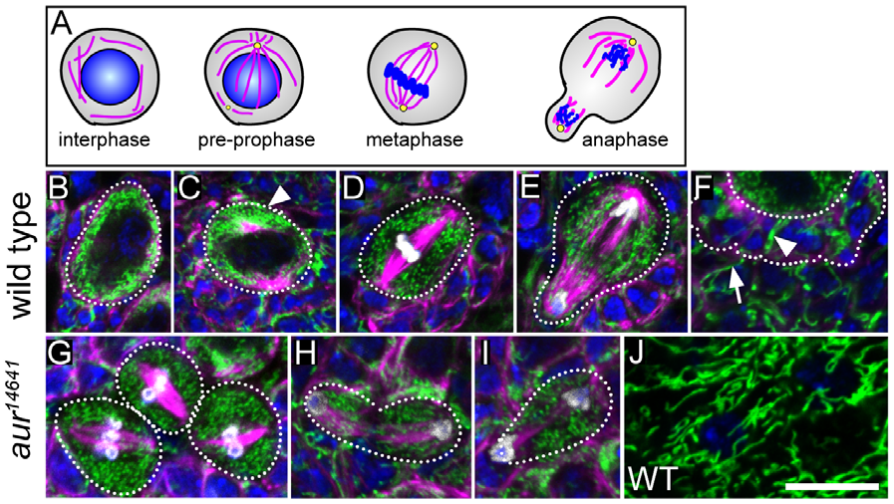
Mitochondria are abundant, small spheres in neuroblasts. A) Cartoon depicting microtubule organization (magenta), centrosomes (yellow) and DNA (blue) during the NB cell cycle. (B–E) Mitochondria during the NB cell cycle. B) During interphase, mitochondria are evenly dispersed in the cytoplasm, and small or slightly oblong. C) The majority of mitochondria aggregate around the first apical aster that forms before mitosis begins. D) Once the spindle forms mitochondria are evenly dispersed around the cell periphery and exclusively small spheres. E) During anaphase, only a small number of mitochondria segregate into the developing GMC. F) In contrast to the NB, the GMCs (dotted outline) have longer mitochondria (arrowhead). The surrounding glia have even longer mitochondria compared to either NBs or GMCs (arrow). (G–I) Mitochondria in *aur^14641^* mutant NBs. G) Mitochondrial shape is the same during mitosis in *aur^14641^* mutant NBs as wild type. H) In symmetric *aur^14641^* NB divisions, mitochondria appear to be evenly divided. I) This is in contrast to asymmetric *aur^14641^* NB cell divisions, which look similar to wild type. J) Mitochondria are very long and branched in the specialized glia that surround the brain and comprise the blood brain barrier. anti-CVα – green for B–J, microtubules – magenta for B–I, DAPI – blue for B–J, anti-phosphohistone H3 – white for B–J. Error bar  = 10 µm for B–J.

Although *clu* mutants do not have a decreased number of NBs, it was possible that lack of Clu could manifest a subcellular mitochondrial phenotype. Before we could determine if this was the case, we first characterized mitochondrial localization and dynamics in wild type NBs. Third instar NBs are large, round cells relative to the surrounding glia. During the wild type NB cell cycle, mitochondria are stereotypically localized and are a consistent small size (Fig. 2B–E). NB mitochondria are plentiful and do not form a reticulum. During interphase, when the microtubule cytoskeleton is randomly arrayed throughout the cytoplasm (Fig. 2A), mitochondria remain evenly dispersed and mostly small and spherical, although some are slightly longer ovals (Fig. 2B). This pattern of even dispersal only changes when there is an obvious, large aster of microtubules at the apical cortex, presumably emanating from the “activated” centrosome (Fig. 2A, preprophase, C, arrowhead, [19], [20]). At this time during the cell cycle, the majority of the mitochondria aggregate around the dominant centrosome where the microtubules are highly concentrated. This pattern changes once the single, large microtubule aster is replaced by the forming mitotic spindle. At this time, the small spherical mitochondria again disperse evenly around the cell (Fig. 2D), and remain in this pattern through anaphase (Fig. 2E). As the asymmetric NB division results in a GMC containing the short end of the spindle and much less cytoplasm, very few of the mitochondria go into the GMC, with most remaining in the large NBs during cell division (Fig. 2E). *aur* mutant NB do not appropriately specify the NB division plane because the Miranda crescent is randomly placed along the cell cortex, instead of being positioned basally next to the older GMCs. This results in both symmetric and asymmetric cell divisions depending on whether the spindle randomly forms at right angles to the localized Miranda crescent [14], [18]. Mitochondria in *aur^14641^* mutant NBs are normally dispersed during both metaphase and anaphase (Fig. 2G–I). If the division is symmetric, a larger amount of mitochondria segregate with the greater cytoplasmic volume (Fig. 2H). In the same mutant, if the division is asymmetric, mitochondrial segregation looks the same as wild type (Fig. 2I). Thus, the number of mitochondria that end up in the GMC appears to depend on spindle placement and the amount of cytoplasm that segregates to the GMC.

**Figure 3:**
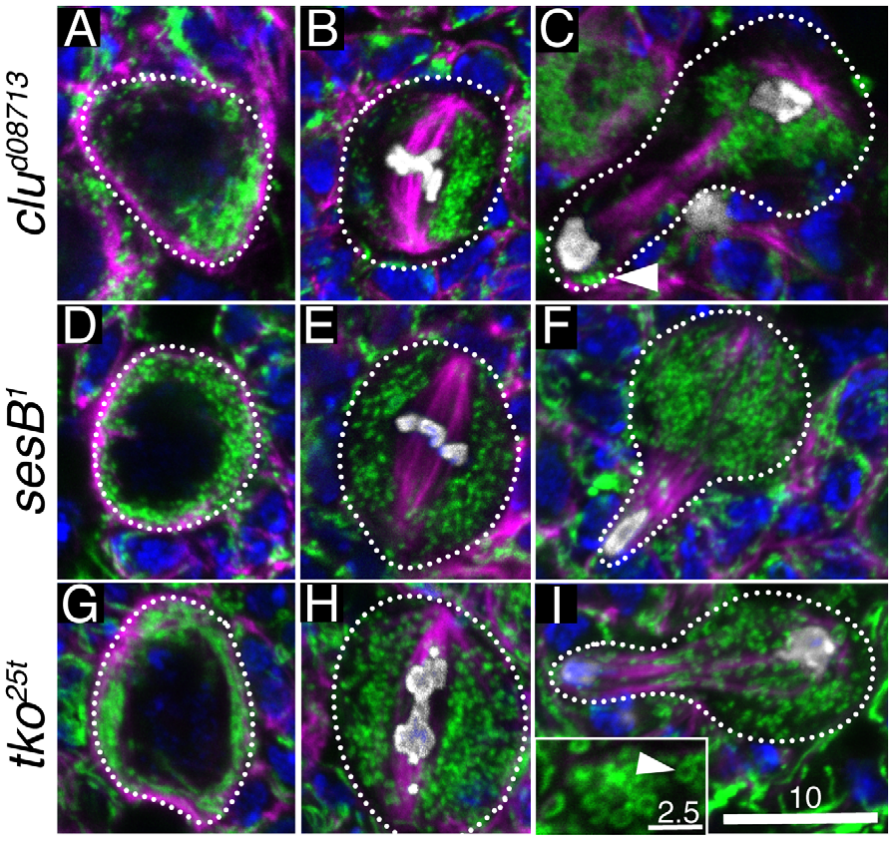
*clu* mutants cause mitochondrial mislocalization in NBs. (A–C) Mitochondrial localization in *clu^d08713^* mutant NBs. A) During interphase, mitochondria clump. B) Abnormal mitochondrial clustering continues during mitosis. C) At anaphase, while mitochondria are still not normally dispersed, a few make it into the GMC (arrowhead). (D–F) Mitochondrial localization in *sesB^1^* mutant NBs. A) Mitochondria are localized normally during interphase (D), metaphase (E) and anaphase (F). During interphase (A), however, the mitochondria are consistently small and fragmented in contrast to wild type. (G–I) Mitochondrial localization in *tko^25t^*. During interphase (G), mitochondria are evenly distributed around the cell periphery, but can be longer than wild type. Metaphase (H) and anaphase (I) have normal mitochondrial localization as well. However, during all phases of the cell cycle, a proportion of mitochondria in the NBs look round and swollen (I, inset, arrowhead). CVα – green, microtubules – magenta, DAPI – blue, phosphohistone H3 – white. Error bars: 10 µm in I for A–I, 2.5 µm in I inset.

While mitochondria are small in NBs, mitochondria in separated GMCs elongate (Fig. 2F, arrowhead). There is an even greater difference in mitochondria length between NB/GMCs and the surrounding glia (Fig. 2F, arrow). Mitochondria are particularly long and branched in the specialized glia that make up the blood brain barrier surrounding the larval brain (Fig. 3J). This is a striking example of how mitochondrial dynamics, including fission and fusion, differs greatly between cell types in vivo.

### Neuroblast Mitochondria in *clueless* Mutants are Mislocalized

**Figure 4:**
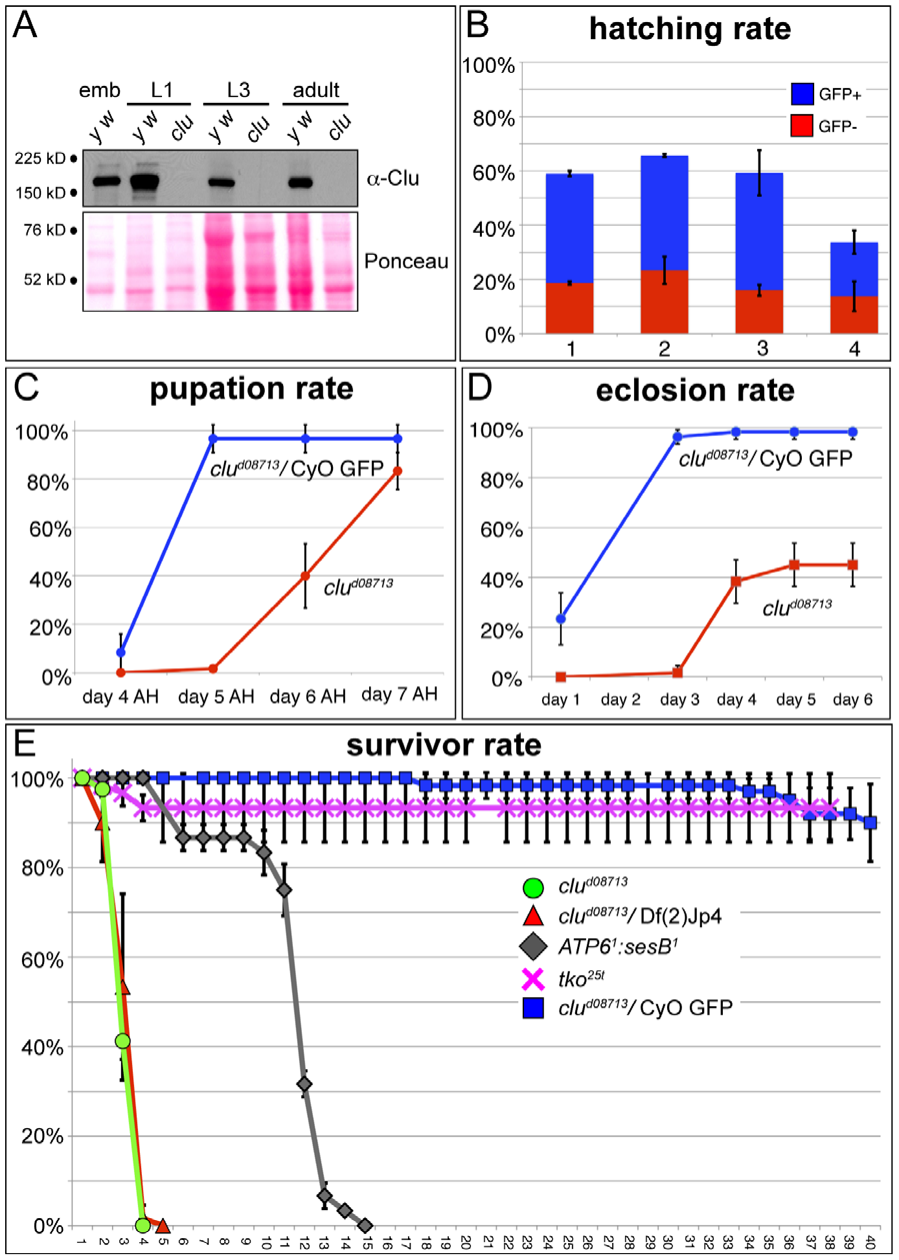
Clu function is critical in adults, but not in larvae. A) Western blot showing Clu is maternally deposited into wild type eggs (*y w*), but is absent in *clu^d08713^* mutants in 1st instar larvae, 3rd instar larvae and adults. emb  = 0–2 hr embryos, L1 =  first larval instar, L3 =  third larval instar. B) Hatching rates. *clu* mutant embryos hatch at normal rates regardless of maternal Clu contribution. Each bar represents hatching rates from progeny of the following parental crosses: 1) +/CyO GFP x +/CyO GFP, 2) *clu^d08713^*/CyO GFP x *clu^d08713^*/CyO GFP, 3) *clu^d08713^*/CyO GFP X Df(2)Jp4/CyO GFP, 4) *clu^d08713^*/*clu^d08713^* (germline clone) x *clu^d08713^*/CyO GFP. C) Pupation rates. *clu^d08713^* mutant larvae experience delayed pupation, but ultimately pupate at numbers comparable to controls. AH = after hatching. D) Eclosion rates. Only 40% of *clu^d08713^* pupae eclose after a 2–3 day delay. E) Survivor curve. *clu^d08713^* homozygous and hemizygous mutant adults die after 3–4 days.

*clu* mutant female germ cells have severe mitochondrial mislocalization [8]. Thus we wanted to examine the mitochondria in *clu* mutant NBs to see if they had a similar phenotype. Throughout the NB cell cycle, mitochondria in *clu^d08713^* mutant NBs are mislocalized and clumped, similar to female germ cells (Fig. 3A–C). Mitochondria in *clu^d08713^* mutant NBs form one or two mitochondrial clumps 87% of the time (n = 90 NBs). In contrast, 53% of wild type NBs have a single mitochondrial clump (n  = 118 NBs), which is when they are normally clustering around the first activated centrosome. It is possible *clu* mutants have a cell cycle arrest right before prophase that results in mitochondrial mislocalization, however, this does not appear to be the case because the mitochondrial clumps are often not near a large microtubule aster, and there is not a large delay in larval development (see Fig. 4). Mitochondrial mislocalization continues throughout the cell cycle in both metaphase (Fig. 3B, 77% (n = 16)) and anaphase (Fig. 3C). Although in anaphase there can be a large, prominent cluster of mitochondria in the NB, some mitochondria still go into the GMC (Fig. 3C, arrowhead). As we have previously found during female germ cell mitosis and oocyte inheritance, as long as a small number of mitochondria segregate into the daughter cell, there appears to be a sensing mechanism that fixes any deficits, likely by increasing mitochondrial replication [8], [21]. Because mitochondria mislocalize in *clu^d08713^* mutant NBs, it is difficult to tell if they are reduced in volume. To determine if there is any reduction, we calculated the volume of mitochondria with a method we have previously used and there does not appear to be any difference in the total mitochondrial volume in *clu^d08713^* mutant NBs compared to wild type (Fig. S2F, Supplementary Materials and Methods, [22]). Thus, even though mitochondria mislocalize throughout the cell cycle in *clu^d08713^* mutant NBs, this does not appear to affect NB number in the central brain, mitochondria volume, or cause gross morphological changes in cell differentiation.

*clu^d08713^* is not just important for mitochondrial localization, but is also important for mitochondrial function. In *clu* mutant germline cells, mitochondria are swollen and lose inner membrane [8]. We wanted to know if mitochondrial mislocalization in *clu^d08713^* mutant NBs was specific for *clu*, or whether other mutants that cause a decrease in mitochondrial function can also cause the same phenotype. We chose to examine mutations in two genes encoding mitochondrial proteins, *stress-sensitive B* (*sesB*) and *technical knockout* (*tko*). *sesB* encodes an adenine nucleotide translocase, which is the protein that exchanges ADP and ATP across the mitochondrial inner membrane [16]. *sesB* is an essential gene, however, we chose to examine *sesB^1^*, which is bang sensitive, because this allele is viable, has a reduced lifespan, and has reduced mitochondrial function [23]. This mutant stock also maintains an additional mutation in its mtDNA-encoded *ATP6* gene that contributes to decreased mitochondrial function [17]. In contrast to *clu^d08713^* mutant NB, *sesB^1^* mutant NBs have normal mitochondrial distribution (Fig. 3D–F). However, these mitochondria are consistently small and spherical throughout the cell cycle compared to wild type or *clu^d08713^* (Fig. 3A vs. D). A similar mitochondrial shape change has been observed in the cells of *sesB^1^* adult renal tubules [24]. *tko* is a nuclear gene that encodes for ribosomal protein S12 of the mitochondrial ribosome [25]. We examined mitochondria in NBs of *tko^25t^*, a bang sensitive *tko* allele that also has reduced mitochondrial function [15], [23]. As with *sesB^1^* mutants, *tko^25t^* mutant NBs have normal mitochondrial localization unlike the severe mitochondrial mislocalization phenotype of *clu^d08713^* mutant NBs (Fig. 3G–I). In contrast to *sesB^1^*, mitochondria are not as fragmented during interphase, and may be slightly longer (Fig. 3G). While mitochondria are dispersed normally during the rest of the cell cycle (Fig. 3H, I), they can be doughnut shaped (Fig. 3I, inset, arrowhead). This shape is usually due to mitochondrial swelling from damage, however, we cannot rule out the possibility that there are defects in mitochondrial fission/fusion.

### Clu Function is Critical for Adults but not Larval Development

Clu protein is highly expressed in larval NBs. In addition, *clu^d08713^* mutant NBs share the conserved mitochondrial mislocalization phenotype associated with lack of *clu* in other Drosophila tissues and model organisms. *clu* mutant adults are very sick, and as we previously showed, only approximately five percent of *clu^d08713^* mutants are present in any given culture [8]. Thus, we were surprised that lack of *clu* does not have a greater impact on larval brain development and, specifically, NBs. To investigate why this is the case, we carefully analyzed *clu* mutants for defects during development. One possibility is that a maternal contribution compensates for zygotic *clu* deficits during larval development. There are high levels of Clu protein in 0–2 hr embryos, indicating that there is a large maternal deposition (Fig. 4A). This is in agreement with large-scale Drosophila transcriptome and *in situ* analyses [26], [27], [28], [29]. However, there is no Clu present in *clu^d08713^* mutant newly hatched 1st instar larvae or 3rd instar larvae (Fig. 4A), indicating any maternal contribution is used up during embryogenesis. Supporting this observation, immunofluorescence does not detect any Clu protein in *clu^d08713^* mutant 3rd instar brains (Fig. 1J).

To definitively rule out the possibility that very low levels of maternal Clu that are undetectable by Western or immunofluorescence play a role in larval development, we created *clu^d08713^* mutant germline clones. The hatching rates are similar between *clu^d08713^* maternal^−^ zygotic^+^ and *clu^d08713^* maternal^−^ zygotic^−^ embryos, indicating an embryo with a paternally contributed *clu^d08713^* mutant chromosome develops equally well compared to an embryo with a paternally contributed wild type chromosome (Fig. 4B, compare blue vs red, bar 4). Overall, fewer eggs laid by females containing *clu^d08713^* germline clones hatch (Fig. 4B, bar 4 vs. bar 2, 3). This is likely due to defects in oogenesis as there was a higher percentage of flaccid, small, and off-white eggs that did not look normal compared to wild type (data not shown). Based on this observation, we do not believe that lack of Clu causes substantial defects during embryogenesis, however, we cannot formally rule this out. *clu^d08713^* maternal^−^ zygotic^−^ larvae also have mislocalized mitochondria in their NBs (Fig. S3), but they are able to eclose into adults, again supporting that maternal Clu is not required for larval development (data not shown). We therefore do not believe that maternally deposited Clu perdures during larval development, and thus Clu protein cannot play a significant role in supporting larval development.

*clu^d08713^* mutant larvae pupate at normal rates, albeit by a three day delay (Fig. 4C). However, only 40% of the pupae are able to eclose and the rest die as pharate adults (Fig. 4D). This explains why at any given time in a fly culture, there are very few *clu^d08713^* homozygous mutant adults. In contrast, adult *clu^d08713^* mutants die very quickly after eclosion, as we have observed previously, and *clu^d08713^* mutants die at the same rate as *clu^d08713^* hemizygotes. (Fig. 4E, [8]). Therefore, Clu does not appear to be crucial larval development, but is for pupal development and soon after eclosion.

### Clu Mutant Adults have Increased Oxidative Damage and Greatly Decreased ATP Levels

**Figure 5:**
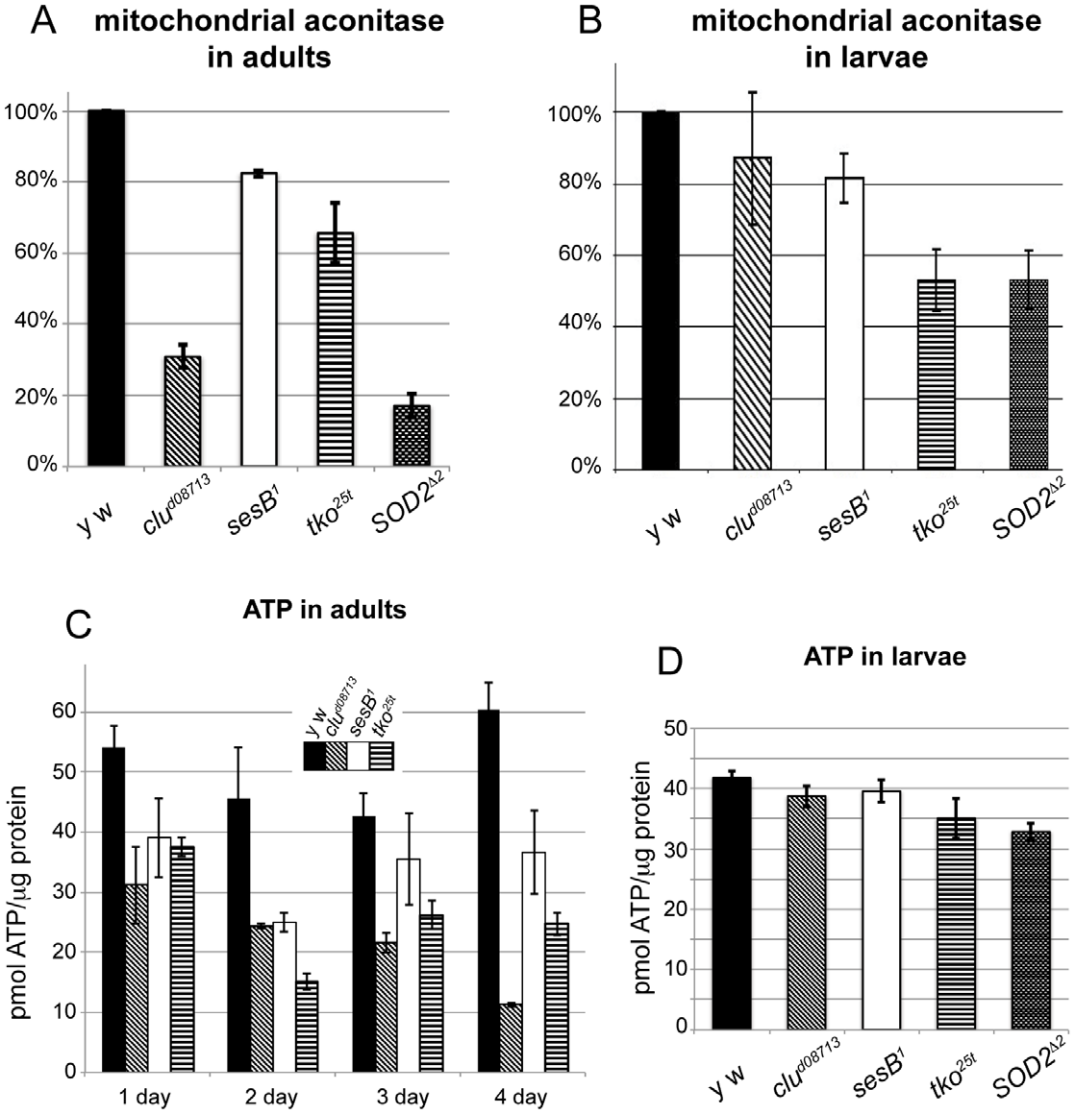
*clu^d08713^* mutant adults, but not larvae, have greatly reduced ATP and increased mitochondrial oxidative damage. A) Mitochondrial aconitase activity in adults. *clu^d08713^* and *SOD2^Δ2^* mutants have greatly increased mitochondrial oxidative damage. B) Mitochondrial aconitase activity in larvae. *clu^d08713^* mutant larvae do not suffer from mitochondrial oxidative damage, in contrast to *tko^25t^* and *SOD2^Δ2^* mutants. C) ATP levels in adults. After eclosion, *clu^d08713^* mutants experience decreased levels of ATP, that continue to go down before they die. *sesB^1^* and *tko^25t^* mutants also have decreases in ATP, but not as severely as *clu^d08713^* mutants. D) ATP levels in larvae. *clu^d08713^* mutant larvae have normal ATP levels.

*clu* appears to be necessary to support mitochondrial function based on transmission electron micrographs showing swollen mitochondria, however, the mechanism by which this occurs is not yet clear. In order to establish that mitochondrial function declines when *clu* is absent, we examined ATP levels and aconitase function. Mitochondrial aconitase acts in the TCA cycle and contains an iron-sulfur cluster that is essential for enzymatic activity. Increased amounts of reactive oxygen species can oxidate the iron-sulfur cluster, rendering the enzyme non-functional [30], [31]. Thus, lack of mitochondrial aconitase activity is used as a proxy of general mitochondrial oxidative damage. In addition, since mitochondria are the main source of reactive oxygen species (ROS), oxidative damage caused by mitochondrial ROS often spills into the cytoplasm causing additional damage to the cell. *clu^d08713^* mutant adults have severely decreased amounts of mitochondrial aconitase activity (Fig. 5A, S4). This indicates mitochondria are experiencing increased oxidative damage. For comparison, mutants for *Superoxidase dismutase 2* (*SOD2*), the enzyme responsible for scavenging free radicals in mitochondria, have decreased amounts of mitochondrial aconitase activity, as has been previously shown [32], [33]. Both *sesB^1^* and *tko^25t^* also have moderately decreased mitochondrial aconitase activity, but neither is as low as *clu^d08713^* nor *SOD2^Δ2^*. In contrast to *clu* mutant adults, *clu^d08713^* mutant larvae do not have greatly diminished mitochondrial aconitase activity, thus lack of *clu* function does not lead to increased amounts of oxidative damage in larvae (Fig. 5B, S4). *sesB^1^* mutants have a modest decrease in mitochondrial aconitase activity, and *tko^25t^* and *SOD2^Δ2^* are decreased by half, indicating these mutant larvae do accumulate oxidative damage (Fig. 5B, S4). These results indicate that while abolishing the superoxide scavenger *SOD2* or the mitochondrial ribosomal protein *tko^25t^* does increase damage, lack of *clu* does not cause any increase in oxidative damage in larvae.

ATP levels are dramatically reduced in *clu^d08713^* mutant adults, which may be the reason why they die so quickly (Fig. 5C, 4E). In newly eclosed *clu^d08713^* mutant flies, there is already a reduction in ATP, which drops over the following three days. This reduction on day one may be related to why only 40% of *clu* mutants eclose (Fig. 4D). As has been shown previously, both *sesB^1^* and *tko^25t^* adults also have reduced ATP concentrations [23]. In contrast, ATP levels are normal in *clu^d08713^* larvae (Fig. 5D). This is also true for *sesB^1^* mutant larvae. *tko^25t^* and *SOD2^Δ2^* have modest decreases in ATP levels in larvae, which may be related to their increased amounts of oxidative damage. We have also observed that *tko^25t^* larvae appear weaker than wild type larvae, whereas *SOD2^Δ2^* larvae behave normally (data not shown). These data support that Clu’s role in mitochondrial function is required for ATP production in adults. This also supports previous observations that larval metabolism is markedly different from adult metabolism, in that larval ATP production relies on aerobic glycolysis, rather than oxidative phosphorylation [13].

## Discussion

### Mitochondria Remain Fragmented in Larval NBs

These studies are the first to examine mitochondrial dynamics during the Drosophila larval NB cell cycle. We first show that mitochondria remain small and fragmented in larval NBs. One explanation for this phenomenon is that larval NBs undergo rapid mitoses every 25–45 minutes (for examples see [19], [20]). Mitochondria in mitotic female germ cells, which also undergo rapid divisions, remain small and dispersed as well [22]. By remaining small and evenly dispersed in the cytoplasm, mitochondria can more easily segregate to the daughter cell. Comparing female germ cell division and NB division, it appears that spindle placement and segregation of cytoplasm are responsible for mitochondrial segregation during normal mitosis. Whereas normal asymmetric NB divisions segregate only a small number of mitochondria into the GMC, *aur^14641^* mutant NBs undergoing symmetric divisions appear to evenly divide their mitochondria. Similarly, female germ cells undergoing symmetric divisions evenly divide their mitochondria [22].

While NBs contain fragmented mitochondria, the surrounding glia contain elongated and branched mitochondria, particularly in the specialized glia that comprise the blood brain barrier. This observation illustrates that mitochondrial size and shape are different in various tissues *in vivo*, and that mitochondria are not always found in a reticulum.

### Clu is Highly Expressed in Larval NBs

Clu’s expression in the third instar brain shows that NBs and all dividing regions of the brain have distinctively high levels of Clu and that Clu is exclusively cytoplasmic. This is in contrast to other NB specific genes. Proteins that specifically label NBs are involved in temporal factor switching (e.g. transcription factors found in the nucleus) or asymmetric cell divisions (proteins localized to the plasma membrane). Some additional proteins include Cadherin, Armadillo, and APC2, however these proteins are bound to the plasma membrane [34], [35]. Clu’s high level in NB cytoplasm is consistent with its localization pattern in both germ cells and follicle cells. And like the ovary, Clu is also found in discreet particles in NBs and medullar neuroblasts, suggesting that these particles can be found in many different cells types and may play a biological role in the cell. Thus, Clu is the first cytoplasmic protein involved in mitochondrial function that specifically labels larval NBs.

Cells lacking *clu* mislocalize mitochondria. This is true for Drosophila female germ cells, as well as Dictyostelium, yeast, and Arabidopsis, indicating *clu* is not only conserved in sequence but also in function [8], [9], [10], [11], [12]. We have extended this observation to larval NB. In *clu* mutant NB, mitochondria form one or two clusters of mitochondria during the entire cell cycle. However, they are still able to segregate enough mitochondria to the GMC to allow apparent normal development and growth. This observation is similar to dividing *clu* mutant female germ cells [8]. Mitochondria consistently mislocalize in a clump away from the daughter cell during mitosis, yet even only a few mitochondria in the daughter cell are enough to repopulate the cell. We know that Drosophila germ cells have multiple mechanisms to control the number of mitochondria present, and it appears NBs can also do the same [8], [21].

### Clu Functions to Guard Against Increased Mitochondrial Oxidative Damage

Even though *clu* mutant larval NBs mislocalize their mitochondria, this does not cause loss of NB, or any gross morphological differences in brain development. The presence of a large maternal contribution is not the reason for this observation since we are unable to detect any Clu protein in larval stages, and even *clu* maternal^−^ zygotic ^−^ germline clones are able to develop and eclose. This result was unexpected at first, given how highly Clu is expressed in NB and how sick adult *clu* mutants are. However, *clu* mutant larvae have normal levels of ATP and do not suffer from mitochondrial oxidative damage, in contrast to *clu* mutant adults. This observation agrees with data demonstrating larval metabolism is quite different from adult metabolism. Drosophila larvae appear to primarily undergo aerobic glycolysis, rather than aerobic metabolism [13]. Larvae must increase in size rapidly during a short time period, and thus have different metabolic needs compared to adult flies. This is akin to tumor growth and is similar to the so-called Warburg effect [36], [37]. Thus, even if *clu* mutant NBs have reduced mitochondrial function, this would not be as deleterious in larvae as it is in adults.

The abnormal localization of mitochondria in *clu* mutant NBs is somewhat surprising, given that *clu* mutant larvae have normal levels of ATP and aconitase function. *tko^25t^* mutants do not mislocalize NB mitochondria even though they have an increase in oxidative damage. Thus, it is likely that mitochondrial mislocalization in larval NBs is not due to general oxidative damage, but to some other mitochondrial function that has yet to be revealed. Both *tko^25t^* and *sesB^1^* encode mitochondrial proteins, whereas *clu* encodes a cytoplasmic protein. Clu may be peripherally localized to mitochondria based on the fact that Clu particles in the female germline always associate with mitochondria [8]. Thus, the mitochondrial mislocalization occurring in *clu* mutant larval NBs must be related more specifically to *clu*’s function, which may be upstream of *tko^25t^* and *sesB^1^* function.

*clu* mutant adults die after three to four days, and by day three have greatly reduced aconitase function. We do not presently know if in *clu* mutants oxidative damage leads to loss of ATP, vice versa, or if they are independent of each other. In agreement with our observations that *clu* mutant adults have increased oxidative damage, our previous microarray analysis comparing *clu* mutant follicles to wild type found gene expression was strongly altered in genes that normally function to protect cells against reactive oxygen species [8]. Thus, in adults Clu appears to plays a role to keep oxidative damage in check and to maintain normal levels of ATP.

## Supporting Information

Figure S1 **Clu is highly expressed in dividing cells in the larval brain.** A) Clu is found in the columnar epithelial cells that comprise the laminar furrow (arrow), the inner proliferative center (B, IPC, arrow) and the NBs found in the ventral nerve cord (C, arrow). Clu particles can also be seen in these cells, for example the epithelial cells (D’) that are next to the laminar furrow (D, arrow). anti-Clu- green, DAPI – blue for A–D’, anti-Mir- magenta for A, B, microtubules – magenta for D, D’. Scale bars: 100 µm (C for A–C), 10 µm (D and D’).
(TIF)

Figure S2 ***clu*** **mutant larval brains develop normally.** (A, B) Actin labeled third instar brains. *clu^d08713^* mutant brains (A) have overall normal structure compared to wild type larval brains (B) as judged by actin staining. (C, D) Anti-Prospero antibody labeled differentiating neurons in a similar pattern in *clu^d08713^* mutant (D) and wild type larval brains (C). E) The number of NB in each anterior ventral central brain hemisphere is the same between *clu^d08713^*/CyO and *clu^d08713^* mutants. F) The percent total mitochondrial volume per cell volume is the same between *clu^d08713^* MARCM NB clones and wild type NB clones. Phalloidin – green, DAPI – blue for A, B. anti-Prospero – green, anti-Mir – magenta for C, D. Scale bars  = 100 µm (B for A, B) and (D for C, D).
(TIF)

Figure S3 **Abolishing maternal Clu causes mislocalized neuroblast mitochondria.** A) *clu^d08713^* germline clone, paternally rescued with a balancer chromosome, has normal mitochondrial localization in 1st instar NBs (dotted outline). B) NBs in first instar brains (dotted outline) from *clu^d08713^* germline clone lacking zygotic *clu* have mislocalized mitochondria. CVα – green, phalloidin – magenta, DAPI – blue. Scale bar  = 10 µm.
(TIF)

Figure S4 **Mitochondrial aconitase activity is reduced in** ***clu^d08713^*** **mutant adults.** The top blots show mitochondrial aconitase activity (mito, bottom bands) and cytoplasmic aconitase activity (cyto, top bands) in adults and larvae. As a loading control, the bottom two blots show mitochondrial aconitase activity reactivated by a reducing agent and ferrous ammonium sulfate. Plus = anode, minus = cathode.
(TIF)

Materials and Methods S1 (DOCX)
