# Supplementary figures and images for: Drosophila Clueless Is Highly Expressed in Larval Neuroblasts, Affects Mitochondrial Localization and Suppresses Mitochondrial Oxidative Damage
Source: PLoS One. 2013 Jan 16;8(1):e54283. doi: 10.1371/journal.pone.0054283 (PMC3547001; doi:10.1371/journal.pone.0054283)

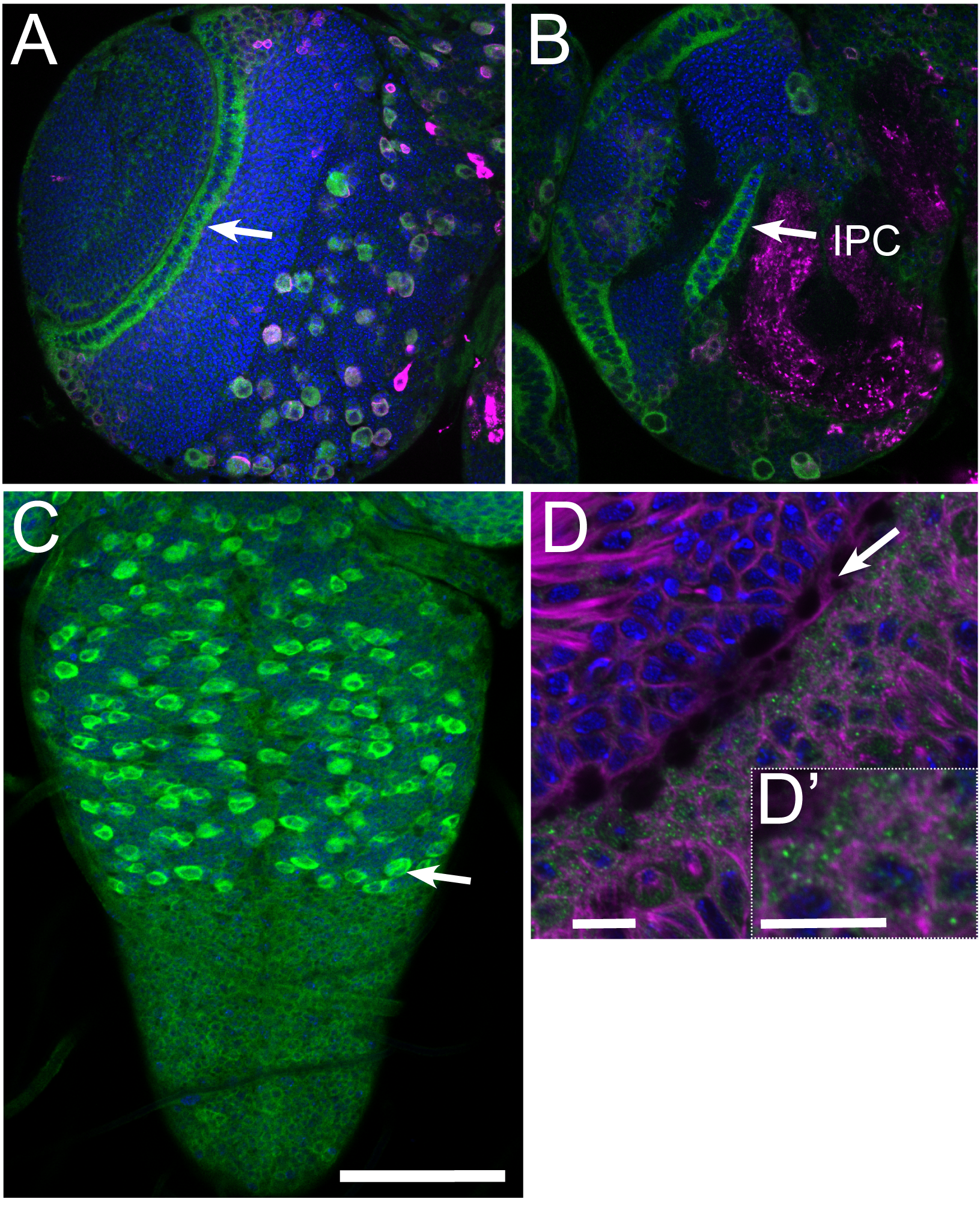

Supplement: Figure S1 — Clu is highly expressed in dividing cells in the larval brain. A) Clu is found in the columnar epithelial cells that comprise the laminar furrow (arrow), the inner proliferative center (B, IPC, arrow) and the NBs found in the ventral nerve cord (C, arrow). Clu particles can also be seen in these cells, for example the epithelial cells (D’) that are next to the laminar furrow (D, arrow). anti-Clu- green, DAPI – blue for A–D’, anti-Mir- magenta for A, B, microtubules – magenta for D, D’. Scale bars: 100 µm (C for A–C), 10 µm (D and D’). (TIF) [file pone.0054283.s001.tif]

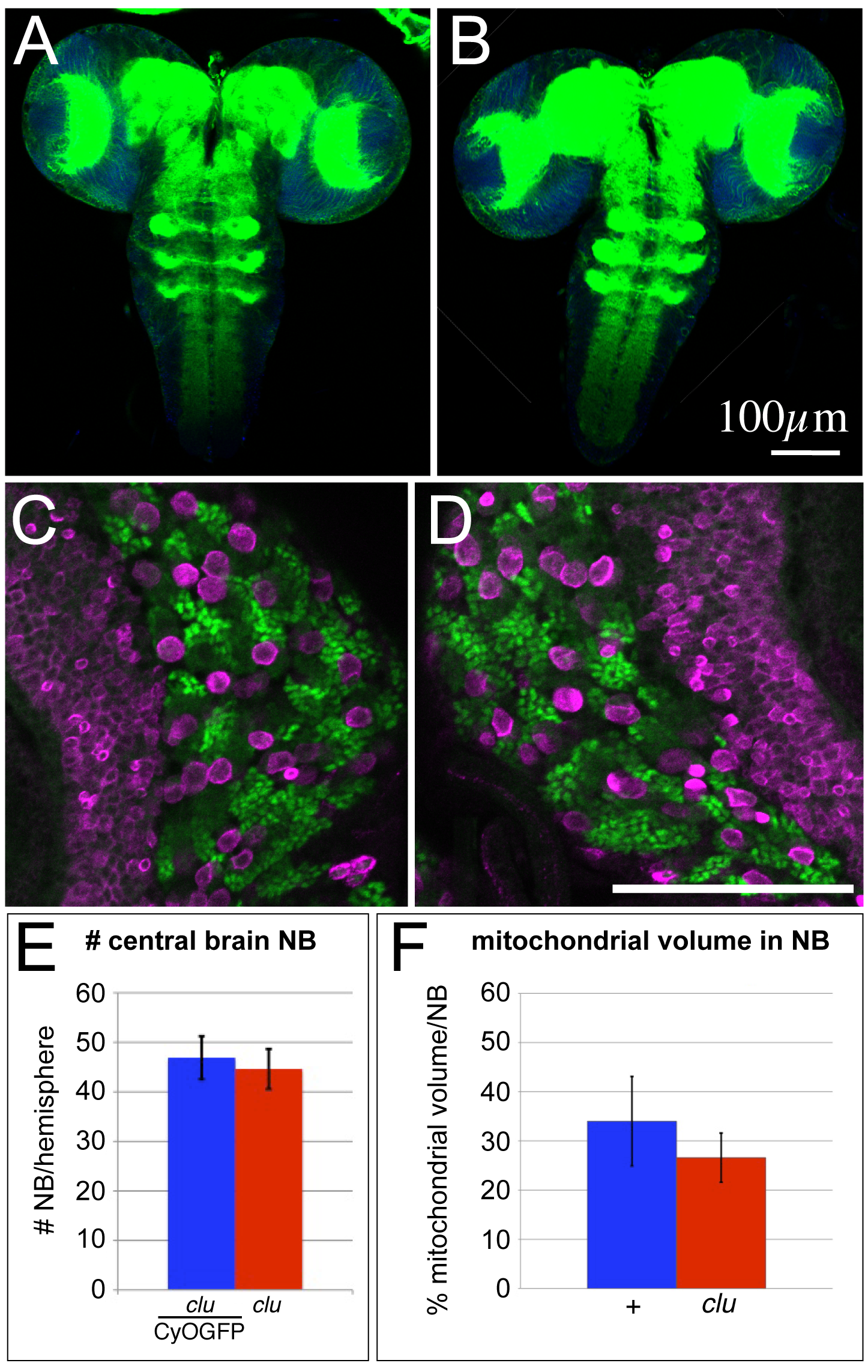

Supplement: Figure S2 — clu mutant larval brains develop normally. (A, B) Actin labeled third instar brains. clud08713 mutant brains (A) have overall normal structure compared to wild type larval brains (B) as judged by actin staining. (C, D) Anti-Prospero antibody labeled differentiating neurons in a similar pattern in clud08713 mutant (D) and wild type larval brains (C). E) The number of NB in each anterior ventral central brain hemisphere is the same between clud08713/CyO and clud08713 mutants. F) The percent total mitochondrial volume per cell volume is the same between clud08713 MARCM NB clones and wild type NB clones. Phalloidin – green, DAPI – blue for A, B. anti-Prospero – green, anti-Mir – magenta for C, D. Scale bars = 100 µm (B for A, B) and (D for C, D). (TIF) [file pone.0054283.s002.tif]

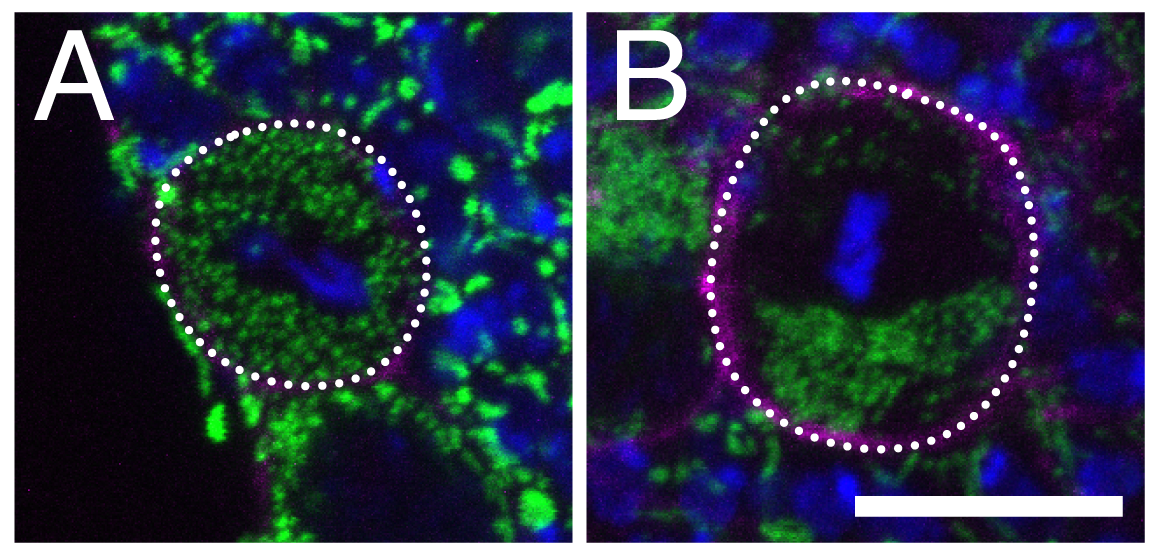

Supplement: Figure S3 — Abolishing maternal Clu causes mislocalized neuroblast mitochondria. A) clud08713 germline clone, paternally rescued with a balancer chromosome, has normal mitochondrial localization in 1st instar NBs (dotted outline). B) NBs in first instar brains (dotted outline) from clud08713 germline clone lacking zygotic clu have mislocalized mitochondria. CVα – green, phalloidin – magenta, DAPI – blue. Scale bar = 10 µm. (TIF) [file pone.0054283.s003.tif]

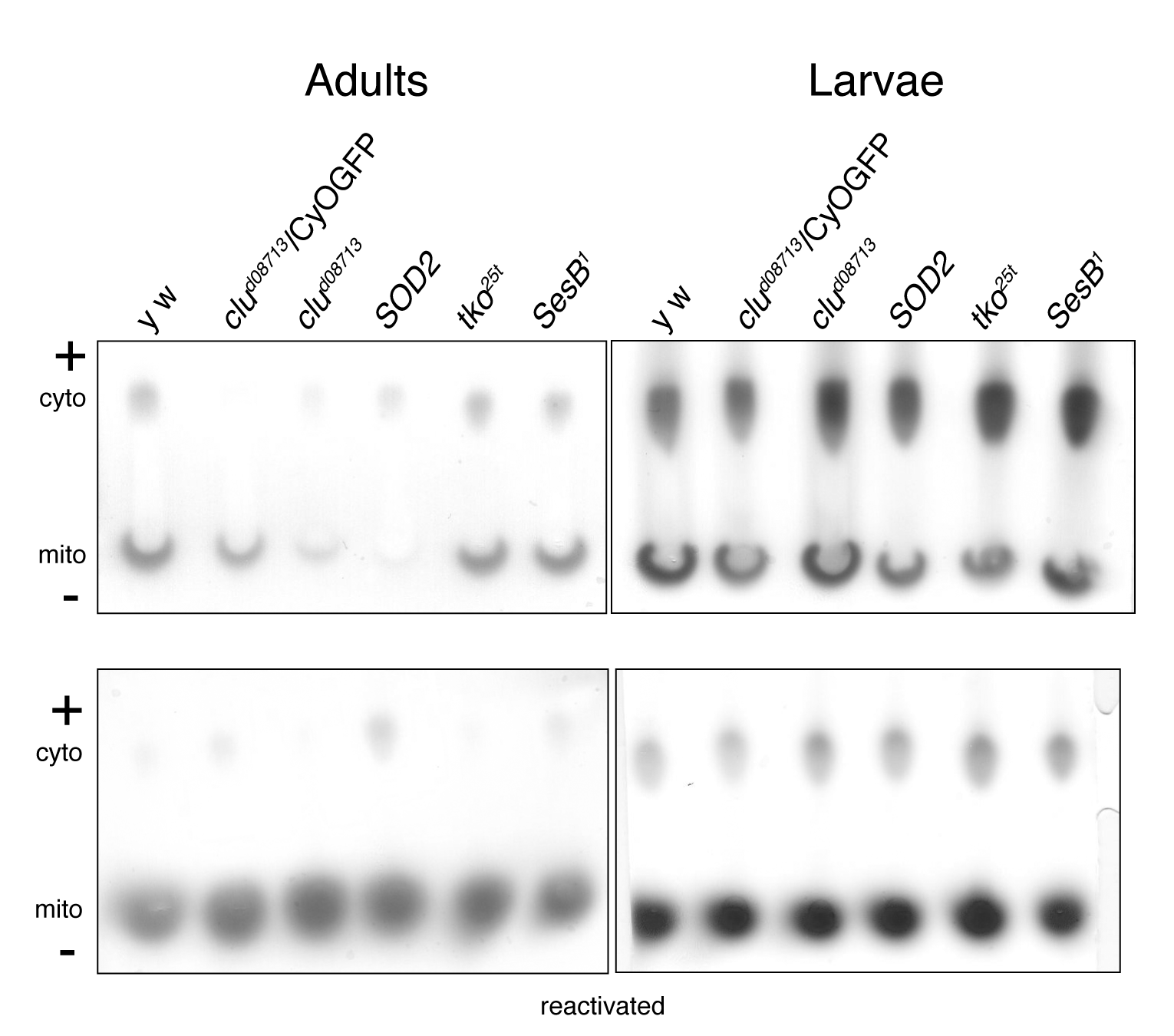

Supplement: Figure S4 — Mitochondrial aconitase activity is reduced in clud08713 mutant adults. The top blots show mitochondrial aconitase activity (mito, bottom bands) and cytoplasmic aconitase activity (cyto, top bands) in adults and larvae. As a loading control, the bottom two blots show mitochondrial aconitase activity reactivated by a reducing agent and ferrous ammonium sulfate. Plus = anode, minus = cathode. (TIF) [file pone.0054283.s004.tif]
