# Supplementary material for: Drosophila Clueless Is Highly Expressed in Larval Neuroblasts, Affects Mitochondrial Localization and Suppresses Mitochondrial Oxidative Damage
Source: PLoS One. 2013 Jan 16;8(1):e54283. doi: 10.1371/journal.pone.0054283 (PMC3547001; doi:10.1371/journal.pone.0054283)
Supplement: Materials and Methods S1 — (DOCX) [file pone.0054283.s005.docx]

Supplementary Materials and Methods:

***Neuroblast numbers:*** Brains were dissected from *clu^d08713^* mutant wandering third instars or *y^1^ w^67c23^* controls and labeled with rhodamine phalloidin and DAPI. Using ImageJ (NIH freeware) each neuroblast was identified per hemisphere in 18 brains of each genotype by its distinctive round shape, attached cluster of GMCs, and diffuse DAPI stain. Eighteen hemispheres were counted for each genotype.

***Neuroblast mitochondrial volume:*** We created *clu^d08713^* MARCM clones and wild type labeled MARCM clones as follows: *w*, *elav*-Gal4, UAS-mCD8-GFP, hs-flp; FRT42D, *tub*-Gal80/CyoKrGFP females were crossed to FRT42D *clu^d08713^*/CyoKrGFP or FRT42D/CyoKrGFP males. Larvae were heat shocked at 37°C for 1 hour 3 times at 48, 72 and 96 hours after egg laying. Third instar larvae were dissected and fixed as in the Materials and Methods section, labeled with mouse anti-Complex V alpha subunit (1:1000, Mitosciences, Inc), rhodamine phalloidin (1:200, Molecular Probes, Invitrogen), rabbit anti-GFP (1:2000, Torey Pines, Inc), followed by anti-mouse IgG_2b_ Alexa 488, anti-rabbit Alexa 633 (Molecular Probes, Invitrogen) and DAPI, then imaged with optimal sectioning on the confocal in the green channel (mitochondria). Using ImageJ, each section for ten NBs of each genotype was outlined using the free hand tool based on phalloidin labeling to calculate the cell volume. To calculate mitochondrial volume, for each section we chose the optimal threshold that best represented the amount of mitochondrial signal, and using the same threshold for every section, converted the section into binary, then calculated the number of pixels labeled. The percentage mitochondrial per cell volume was then calculated.
